# Long-Term Follow-Up of SAPHO Syndrome for 15 Years Led to a Diagnosis of Temporomandibular Joint Pain and Trismus

**DOI:** 10.1155/2021/3102037

**Published:** 2021-11-26

**Authors:** Koki Takamatsu, Hitoshi Sato, Takashi Moriya, Arisa Yasuda, Takaaki Kamatani, Tatsuo Shirota

**Affiliations:** ^1^Department of Oral and Maxillofacial Surgery, School of Dentistry, Showa University, Tokyo, Japan; ^2^Department of Oral Oncology, School of Dentistry, Showa University, Tokyo, Japan

## Abstract

Synovitis, acne, pustulosis, hyperostosis, and osteitis (SAPHO) syndrome is a systemic disease with symptoms of pustular skin disease and sterile osteoarticular lesions. This disease rarely involves the temporomandibular joint (TMJ). Although it is a disease with a good long-term prognosis, its treatment remains challenging. We describe a case with long-term follow-up of SAPHO syndrome for 15 years in which TMJ pain and trismus led to the diagnosis. A 30-year-old woman with TMJ pain and trismus was referred to our department. Her medical history included palmoplantar pustulosis. Sterile inflammation in the left TMJ and diffuse sclerosing osteomyelitis of the mandible were observed. Thus, she was diagnosed with SAPHO syndrome. The symptoms of severe TMJ pain, trismus, and left cheek swelling presented three times in the 15 years. Symptomatic treatment with nonsteroidal anti-inflammatory drugs, antibiotics, corticosteroids, and bisphosphonates was administered several times. There has been no relapse of symptoms over the past nine years. The patient must be continuously kept under observation to look for the relapse of symptoms.

## 1. Introduction

Synovitis, acne, pustulosis, hyperostosis, and osteitis (SAPHO) syndrome causes inflammatory changes in the bones, joints, and skin. It is a systemic disease manifested by pustular skin disease, aseptic bone, and joint lesions [1]. In particular, bone and joint lesions are classified as osteomyelitis, periostitis, and arthritis. Diffuse sclerosing osteomyelitis of the mandible (DSOM) occurs in approximately 10% of cases of SAPHO syndrome but rarely involves the temporomandibular joint (TMJ) [2].

Usually, symptomatic treatment is provided for bone and joint lesions. Nonsteroidal anti-inflammatory drugs (NSAIDs) such as indomethacin and diclofenac sodium are effective [3, 4]. Although SAPHO syndrome causes aseptic inflammation, secondary infections may occur from nearby tissues, and antibiotics such as azithromycin and doxycycline are mainly used. Steroid therapy has also been reported to be effective, but the details are unknown. Indications of antibacterial agents and bisphosphonates for cases that are difficult to control with NSAIDs may be considered [5]. Here, we report the case of a patient with SAPHO syndrome who was treated with NSAIDs, antibacterial drugs, corticosteroid hormones, and bisphosphonates and was followed up for a long period.

## 2. Case Presentation

**Figure 1:**
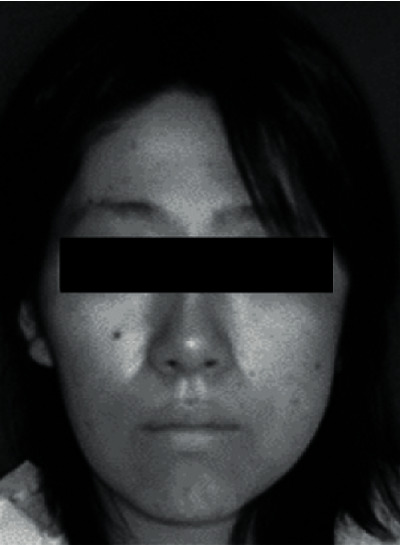
A slight bulge is observed from the left cheek to the lower jaw.

The patient was a 30-year-old woman. Her medical history included palmoplantar pustulosis. The first consultation was in September 2004, and the chief complaint was left TMJ pain. Extraoral findings showed tenderness in the left TMJ and pain at the time of opening. The maximum painless opening is 13 mm. The facial appearance was asymmetrical, and a slight bulge was observed from the left cheek to the lower jaw, but no major abnormalities were observed in the oral cavity (Figure 1).

**Figure 2:**
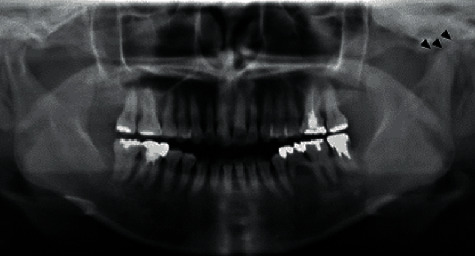
The panoramic radiograph showing absorption changes in the left mandibular condyle (arrowhead).

**Figure 3:**
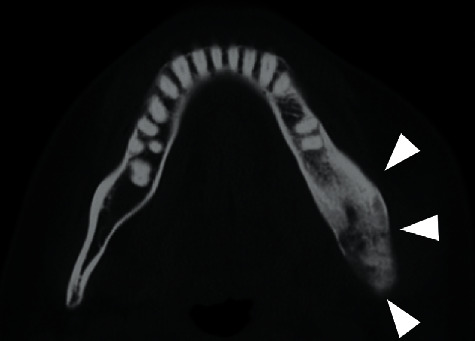
Computed tomography showing a bulge with an osteosclerosis was observed from the left mandibular branch to the mandibular condyle (arrowhead).

The panoramic radiography findings showed absorptive changes of the left mandibular condyle (Figure 2). Computed tomography (CT) findings showed swelling in the buccal side of the mandibular bone and sclerosing changes in the bone marrow from the body to the ramus of the left mandible (Figure 3). Based on these results, we diagnosed left mandibular osteomyelitis and associated trismus.

### 2.1. Treatment Time and Course

#### 2.1.1. 2004

Although the dental infection was suspected as the primary disease of left mandibular osteomyelitis, the cause of infection could not be identified from a panoramic radiograph and oral findings. Administration of NSAIDs (loxoprofen sodium) showed a tendency to improve TMJ pain and trismus, but swelling of the left TMJ persisted. Therefore, fine needle aspiration of the upper joint cavity was performed. Since the synovial fluid was colorless, transparent, and odorless, the patient was diagnosed with noninfectious jaw arthritis. When 3.3 mg of dexamethasone was injected into the upper joint cavity of the left TMJ, pain disappeared completely, and the maximum mouth opening amount increased to 29 mm. Since the patient had a history of palmoplantar pustulosis and CT images showed diffuse sclerotic changes in the left mandible, it was considered to correspond to bone and joint lesions associated with palmoplantar pustulosis according to Benhamou's diagnostic criteria. In addition, since there were no diseases that met the exclusion criteria, the patient was diagnosed with SAPHO syndrome.

#### 2.1.2. 2005

Pain in the left TMJ during jaw movement recurred, and painful swelling of the left TMJ to the cheek was observed. The maximum painless mouth opening is 8 mm. Laboratory test showed a white blood cell count of 5,800/*μ*L and CRP level of 2.1 mg/dL. The condition was diagnosed as relapse of chronic mandibular osteomyelitis associated with SAPHO syndrome, and clindamycin 600 mg/day and cefazolin sodium 1 g/day were administered. In addition, a single intravenous dose of 30 mg disodium pamidronate, a bisphosphonate (BP) preparation, was administered. The swelling of the left cheek and pain of the left TMJ improved, and the maximum painless mouth opening increased to 30 mm.

#### 2.1.3. 2010

**Figure 4:**
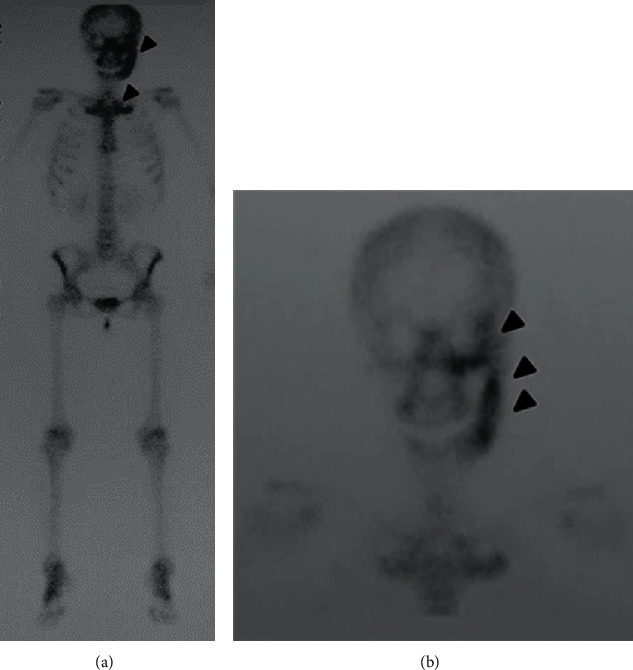
(a) Bone scintigraphy showing abnormal accumulation in the bilateral thoracic rib joints and sternum (arrowhead). It presents with the so-called bull's head sign. (b) Abnormal accumulation is observed in the temporal bone, cheekbone, sphenoid bone, and mandible (arrowhead).

Left TMJ pain and trismus recurred during jaw movement. Laboratory tests showed no abnormal white blood cell count or CRP. Bone scintigraphy showed accumulation of technetium-99m hydroxymethylene diphosphonate (99mTc-HMDP) in the left mandible, including the TMJ (Figure 4(a)), and accumulation in both thoracic rib joints and sternum (Figure 4(b)). In August of the same year, a biopsy of the left mandible was performed under hospitalization and general anesthesia.

**Figure 5:**
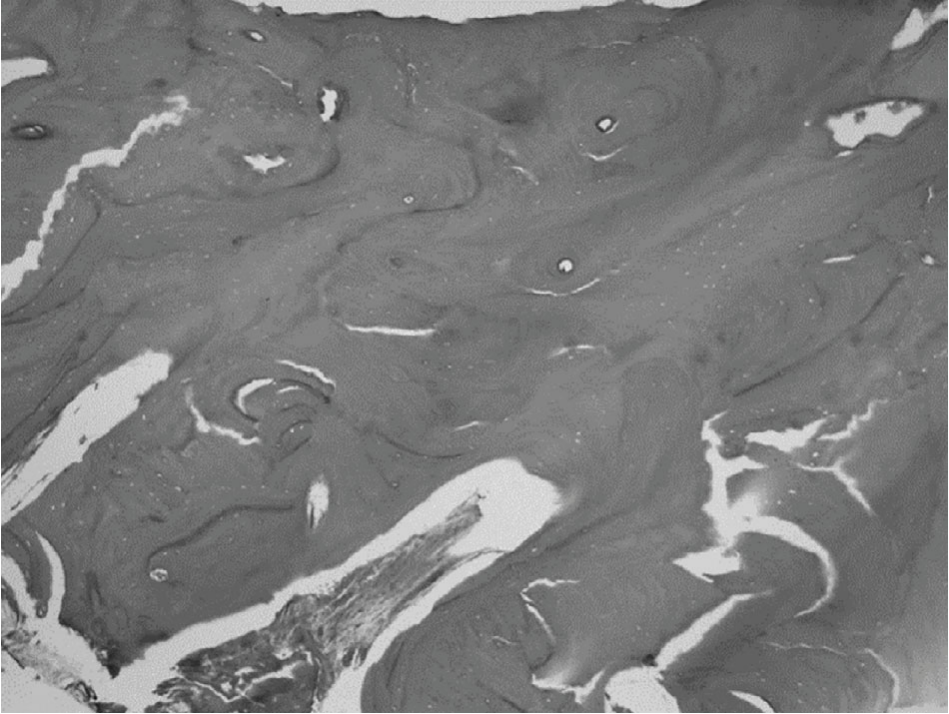
H-E staining showing sclerosing changes and bone cell defects in the trabecular bone.

Pathological findings showed sclerotic changes in the trabecular bone, inflammatory granulation tissue, and fibrous connective tissue with lymphocyte infiltration (Figure 5). Therefore, we made a histopathological diagnosis of sclerosing mandibular osteomyelitis.

### 2.2. Additional Treatment and Course

**Figure 6:**
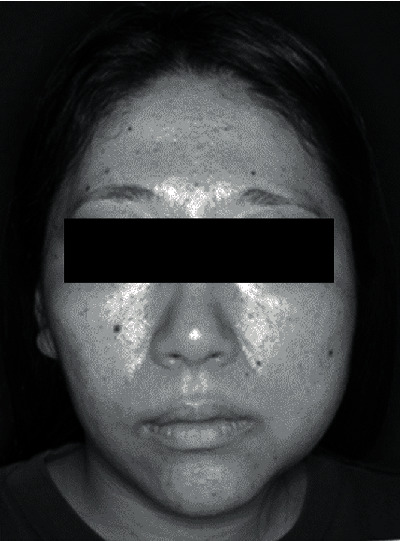
Image showing facial asymmetry with bulging of the left mandible of the patient in 2010.

Hyperbaric oxygen therapy was performed 10 times in total, and clarithromycin 400 mg/day was administered; however, no improvement was observed. Administration of prednisolone 40 mg/day eliminated left TMJ pain and improved trismus. Since then, the patient continued to visit our department regularly, and although his facial asymmetry remained (Figure 6), her symptoms did not recur.

## 3. Discussion

The diagnostic criteria for SAPHO syndrome include (1) bone-related lesions associated with severe acne, (2) bone/joint lesions associated with palmoplantar pustulosis, (3) bone hyperplasia, and (4) chronic recurrent multimyelitis [1]. The mandible is the most common site for bone and joint lesions associated with SAPHO syndrome in the oral and maxillofacial regions. DSOM occurs in approximately 10% of cases of SAPHO syndrome, and most of them are intractable [2, 6, 7]. In this case, it is possible that the inflammation of the DSOM associated with SAPHO syndrome spread to the left mandibular condyle and caused absorptive changes. The affected area rarely included the TMJ since 2010 (Table 1).

Mandibular osteomyelitis associated with SAPHO syndrome needs to be differentiated from purulent mandibular osteomyelitis associated with dental infections [9–11]. This patient had a history of palmoplantar pustulosis, and there was no lesion that could be the source of infection, and resorption of the mandibular condyle and sclerotic changes in the mandible were observed. Therefore, the above diagnostic criteria (2) were met. The applicable exclusion criteria were purulent osteomyelitis, chest wall arthritis due to infection, infectious palmoplantar pustulosis, palmar keratosis, diffuse idiopathic osteoproliferative disorder (DISH), and osteoarthritis associated with retinoid therapy. Therefore, the patient was diagnosed with SAPHO syndrome.

In the treatment, the symptoms improved noninvasively by using drugs such as NSAIDs and BP preparation. In previous reports on nonbacterial osteomyelitis, aggressive surgical treatment resulted in a relapse of symptoms and reports recommending noninvasive treatment [3, 4]. To date, no definitive treatment method has been established, but noninvasive treatment methods such as those using drugs may be effective.

In this case, 15 years have passed since the diagnosis of SAPHO syndrome, and the follow-up period is the longest in the previously reported cases, including the TMJ in the affected area (Table 1). The symptoms did not recur from 2010 to the present. It is necessary to carefully continue the follow-up and consider resection of the mandibular condyle and total replacement of the TMJ when the symptoms recur.

## 4. Conclusion

In this study, we report a case of SAPHO syndrome, which was diagnosed by pain in the left temporomandibular joint and difficulty in opening the mouth and treated with NSAIDs, antimicrobial agents, corticosteroid hormones, and BP preparations, with long-term follow-up. In the results of long-term follow-up, we consider that conservative treatment of osteomyelitis in SAPHO syndrome is preferable to surgical invasion.

## Figures and Tables

**Table 1:** Reported cases of SAPHO syndrome including the temporomandibular joint.

No.	Reporter	Sex	Age	Lesions			Trismus	Swelling	Treatment		Follow-up (month)
				Temporomandibular joint	Chest	Skin			No effect	Effect	
1	Kodama et al. [5] 2013	M	50	○	○	—	○	○	—	NSAIDs, BP, steroids	60

2	Kotaki et al. [8] 2020	M	37	○	○	○	○	—	Antibiotic, coronoid process removal	BP	12

3	Takamatsu et al. 2021	F	30	○	○	○	○	○	Antibiotic	NSAIDs, BP, steroids	180

NSAIDs: nonsteroidal anti-inflammatory drugs; BP: bisphosphonate.

## Abbreviations

SAPHO: Synovitis, acne, pustulosis, hyperostosis, and osteitis
TMJ: Temporomandibular joint
NSAIDs: Nonsteroidal anti-inflammatory drugs.
